# Assessment of the Number and Phenotype of Macrophages in the Human BMB Samples of CML

**DOI:** 10.1155/2016/8086398

**Published:** 2016-11-24

**Authors:** Jian-Xin Song, Zi-Jin Dian, Yan Wen, Fen Mei, Rui-Wei Li, Ya-Lian Sa

**Affiliations:** ^1^Department of Clinical Laboratory, Yunnan Provincial First People's Hospital, Kunming, Yunnan 650032, China; ^2^Institute of Clinical and Basic Medical Sciences, Yunnan Provincial First People's Hospital, Kunming, Yunnan 650032, China; ^3^Department of Hematology, Yunnan Provincial First People's Hospital, Kunming, Yunnan 650032, China

## Abstract

Macrophages have emerged as a key player in tumor biology. However, their number and phenotype in human bone marrow of biopsy (BMB) samples of chronic myeloid leukemia (CML) and their association with disease progression from an initial chronic phase (CP) to accelerated phase (AP) to advanced blast phase (BP) are still unclear. BMB samples from 127 CML patients and 30 patients with iron-deficiency anemia (IDA) as control group were analyzed by immunohistochemistry. The expression levels of CD68, CD163, and CD206 in BMB samples of CML patients were significantly higher than those in the patients of control group (*P* < 0.01), and we observed that their positive expression was gradually elevated during the transformation of CML-CP to AP to BP (*P* < 0.01). However, the expressions of CD68, CD163, and CD206 in released group were downregulated and contrasted to these in control group; there exists statistical significance (*P* < 0.01). The percentage ratio of CD163 and CD206 to CD68 was pronounced to be increasing from CML-CP to AP to BP (*P* < 0.01). Hence, the higher proportion of CD68^+^, CD163^+^ and CD206^+^ macrophages in BMB samples can be considered a key factor for disease progression of CML patients. Targeting macrophages, especially the M2 phenotype may help in designing therapeutic strategies for CML.

## 1. Introduction

Chronic myeloid leukemia (CML) arising from the abnormalities of hematopoietic stem/progenitor cells (HSCs/HSPCs) remains mostly an incurable disease [1]. The stereotypical progression of CML is from a relatively benign chronic phase (CP) to accelerated phase (AP) to a fatal blast phase (BP) resembling acute leukemia, the prognosis for which is poor, with a median survival time of only 3~4 months [2]. Given that all treatments work much better in CP than advanced-phase disease, it is therefore important to explore the mechanism underlying stage progression of CML [3]. Macrophages as critical immune cells and an important member of the bone marrow microenvironments are playing key role in the innate and adaptive immune response involved in tumor biology [4, 5]. Macrophages are very versatile cells with a high degree of plasticity taken on differential phenotype and functions under the physiological and pathological condition provided by local microenvironment. According to two extremes of a spectrum of possible macrophages polarization, macrophages are termed classically activated M1 (proinflammatory type 1) and alternatively activated M2 (anti-inflammatory type 2) subtypes [6, 7]. In the tumor area macrophages have been named tumor-associated macrophages (TAMs) [8, 9]. It was reported that M2-like macrophages are prominently found and involved in cancer initiation, progression, and metastasis, facilitating angiogenesis, matrix breakdown, and tumor cell migration, as well as decreased tumor-infiltrating cytotoxic T lymphocytes (CTLs) [10–13]. However, the TAM counts and its phenotype in the BMB sample of CML patients with different phases are still unsure.

With regard to different responses to various microenvironmental stimuli during CML progression, the count and phenotype of macrophages were considered to be facilitating stage determination and the therapy target. Therefore, we attempted to explore the expression levels of macrophages markers CD68, CD163, and CD206 detected by immunohistochemistry [14–16]. We observed a pronounced increase of CD68^+^, CD163^+^, and CD206^+^ macrophages in the BMB samples of different phases of CML patients. And the percentage ratio of CD163^+^ and CD206^+^ macrophages to CD68^+^ macrophages was upregulated during CML development. Thus, we speculate this may be an important step for the further transformation into AP to BP stages of CML. More importantly, our present data has proposed a novel immunological mechanism for stage progression in CML pathogenesis.

## 2. Materials and Methods

### 2.1. Study Approval

This study was approved by the Medical Ethics Committee of Yunnan Provincial First People's Hospital. Written informed consent was obtained from patients to authorize their participation in the study. Bone marrow biopsies were obtained from recruited adult patients seen at the Department of Hematology.

### 2.2. Patients

We analyzed bone marrow biopsies from 127 patients with chronic myeloid leukemia (CML) in Yunnan Provincial First People's Hospital. These 66 patients with CML received tyrosine kinase inhibitors (TKI) alone or in combination with cytosine arabinoside (Ara-C) or standard anticancer regimens; and 61 patients were followed up for 3 through 15 months.  Table 1 summarizes data related to these patients. The diagnoses of CML were established on the basis of the morphological examination and cytogenesis analysis. The control group was consisted of 30 patients (12 females; 18 males) with iron-deficiency anemia (IDA). The median age was 54.5 years (range, 18–76 years).

### 2.3. Bone Marrow Biopsies (BMB) Samples

Representative bone marrow trephine biopsies were performed from the posterior iliac crest.

### 2.4. Immunohistochemical Analysis

BMB samples were fixed by immersion in 4% buffered formalin and processed overnight at RT. Samples were sequentially decalcified with 10% buffered ethylene-diamine tetra-acetic acid (EDTA), pH 7.2, embedded in paraffin, and then sectioned into 4 *μ*m with a microtome [17]. The slides were baked, deparaffinized in xylene, and rehydrated through a graded alcohols series to water. Antigen retrieval was done by immersing the slides in citric acid buffer solution with pH 6.0 and placed in autoclave at 121°C for 10 min. Washes were done in Tris-buffered saline and 0.05% Tween 20 (pH 7.4). After cooling down to room temperature, sections were treated with 3% hydrogen peroxide (H_2_O_2_) in phosphate-buffered saline (PBS) at room temperature for 30 min. Slides were washed, and blocking was carried out using serum-free protein block at room temperature for 30 min. Slides were incubated with primary antibodies (Abcam company), anti-CD68 (ab955; dilution 1 : 600), anti-CD163 (ab87099; dilution 1 : 600), and anti-CD206 (ab64693; dilution 1 : 600) for detection of macrophages, at 4°C overnight. This was followed by secondary antibody (A biotinylated link antibody and a streptavidin-horseradish peroxidase kit) for 30 min. Finally the slides were washed with PBS and developed with DAB. All sections were counterstained with hematoxylin and then were dehydrated and mounted.

Light microscopy was used to evaluate the intensity and localization of the staining. The positive cell staining with brown in cytoplasm or cell membrane was observed. In each BMB section, a total number of staining cells were viewed and counted by using an objective lens (magnification ×40) and an ocular lens (10x) in at least 5 areas. The numbers of CD68^+^, CD163^+^, and CD206^+^ cells were expressed as percentage. Immunocytochemical staining results were obtained by 2 independent observers.

### 2.5. Statistical Analysis

Data were presented as median (mean ± standard error) and percentage. To determine the level of significance in differences in CD68^+^, CD163^+^, and CD206^+^ numbers between the various groups of CML patients, Kruskal-Wallis and Wilcoxon rank-Wallis test were applied [18]. Statistical analysis was performed using Statistical Package for Social Sciences (SPSS) software (version 17, SPSS, Inc, Chicago, IL, USA). Statistical significance was set at *P* value less than 0.01.

## 3. Results

### 3.1. Evaluation of CD68 in the BMB Samples of CML Patients

**Figure 1:**
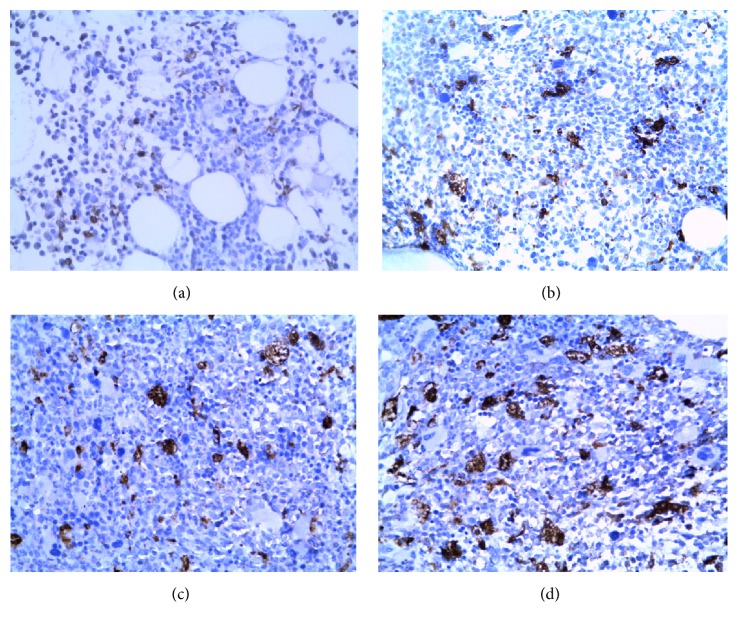
Immunohistochemical staining displaying high numbers of CD68^+^ macrophages in BMB samples of CML patients. CD68 was distributed in the cytoplasm of macrophages (magnification: ×400). Interspersed CD68 expression is shown in control group (a), CML-CP (b), CML-AP (c), and CML-BP (d).

**Figure 2:**
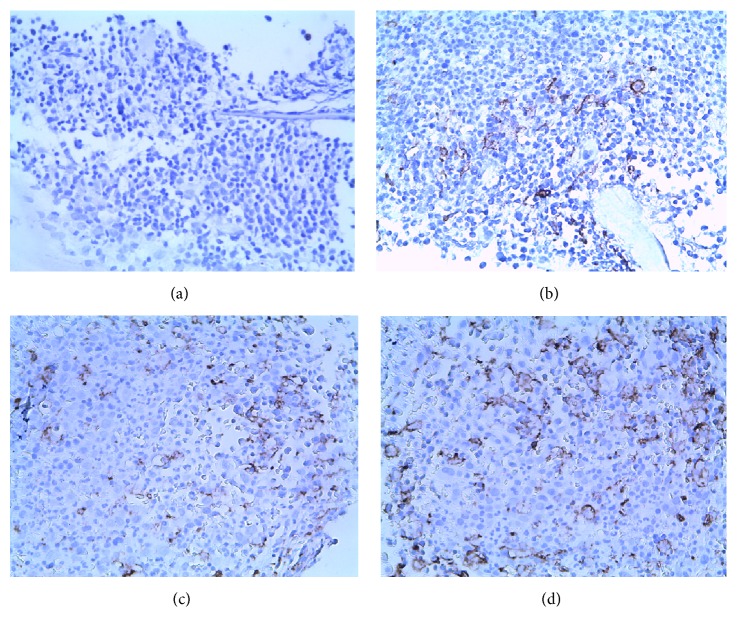
Immunohistochemical staining of CD163 in BMB samples of CML patients. CD163 was located in membrane of macrophages (magnification: ×400). Scatted CD163^+^ macrophages expression is distributed in control group (a), CP-phase (b), AP-phase (c), and BP-phase (d).

CD68^+^ macrophages were roughly satellite shaped with many cytoplasmic processes and revealed a randomly dispersed distribution in the CML bone marrow. The percentage of CD68^+^ macrophages was gradually elevated in BMB samples of CML patients with CML-CP (27.03 ± 3.90)%, CML-AP (44.64 ± 4.84)%, and CML-BP groups (66.98 ± 6.28)% (Figures 1(a), 1(b), 2(c), and 1(d)), respectively. In contrast to the control group (12.39 ± 2.17)%, the CD68^+^ macrophages infiltration density in each one of CML groups was increased significantly higher (*P* < 0.01).

The percentage of CD68^+^ macrophages was decreased in the released patients with CML-CP (19.73 ± 3.43)%, CML-AP (25.77 ± 4.83)%, and CML-BP (43.49 ± 4.80)%, respectively. In contrast to the control group, there existed statistically significant (*P* < 0.01).

### 3.2. CD163 Positive Expression Is Distributed in BMB Samples of CML Patients

The number of CD163^+^ macrophages was remarkably increased in BMB samples from CML-CP (19.91 ± 3.33)% to CML-AP (37.26 ± 4.70)% to CML-BP (59.97 ± 6.79)% (Figures 2(a), 2(b), 2(c), and 2(d)), respectively. The CD163^+^ macrophages infiltration density in BMB samples of CML patients was increased significantly higher than that of the control group (2.71 ± 0.82)% (*P* < 0.01). The expression of CD163^+^ macrophages is distributed in released patients with CML-CP (8.73 ± 2.00)%, CML-AP (17.23 ± 3.24)%, and CML-BP groups (34.11 ± 4.95)%, respectively. In contrast to the control group, there existed statistically significant (*P* < 0.01).

### 3.3. A CML Biopsy Expressed CD206 Was Upregulated

**Figure 3:**
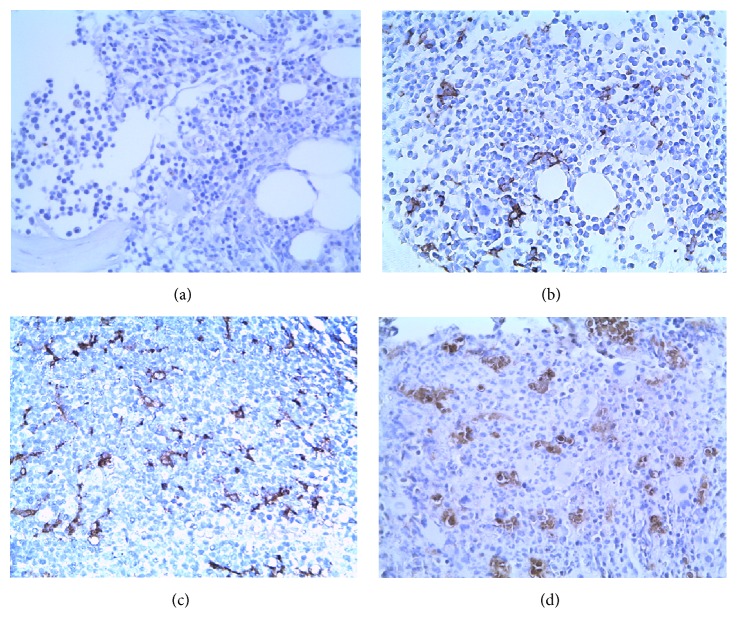
Immunohistochemical staining for CD206 in BMB samples of CML patients (magnification: ×400). Interspersed CD206^+^ macrophages expression is distributed in control group (a), CP-phase (b), AP-phase (c), and BP-phase (d).

The percentage ratios of CD 206^+^ macrophages infiltrating in the CML-CP (20.71 ± 3.47)%, CML-AP (38.57 ± 5.12)%, and CML-BP groups (61.51 ± 6.48)% were dramatically upregulated (Figures 3(a), 3(b), 3(c), and 3(d)), respectively. Compared with the control group (2.94 ± 0.79)%, there existed statistically significant (*P* < 0.01). The percentage of CD206^+^ macrophages was decreased in the released patients with CML-CP (9.03 ± 2.01)%, CML-AP (17.24 ± 3.27)%, and CML-BP groups (34.04 ± 5.06)%, respectively. Contrasted to the control group, there existed statistically significant (*P* < 0.01).

### 3.4. Comparisons of the Staining with CD68^+^, CD163^+^, and CD206^+^ Macrophages Were Shown in the BMB Samples of CML Patients

**Figure 4:**
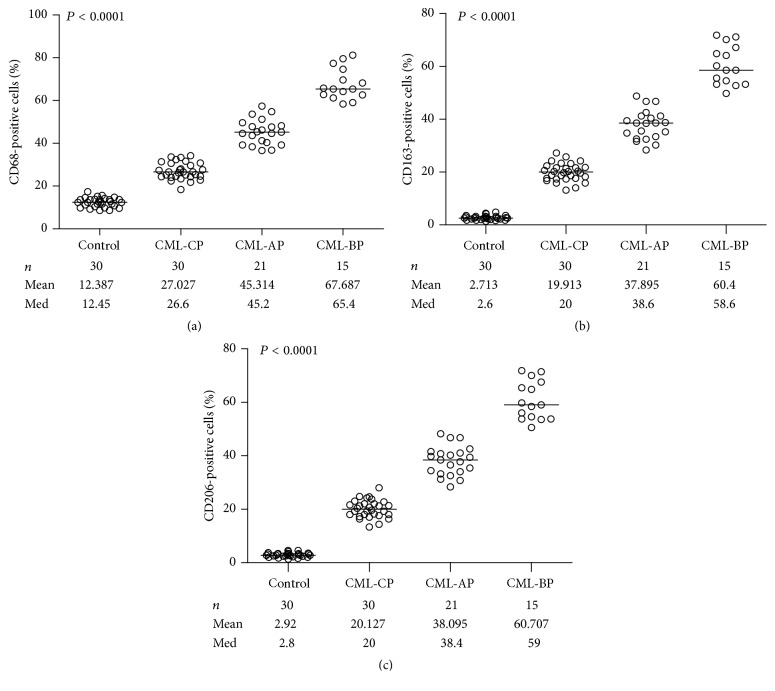
Analysis of infiltrating macrophages in BMB samples of CML patients. The percentage of macrophages positive for CD68 (a), CD163 (b), and CD206 (c) correlated with the development of CML.

The expression levels of CD68, CD163, and CD206 in the BMB samples of CML patients were significantly increased in different phases with disease progression, especially in the CML-BP group which was infiltrated with highest frequency of macrophages (Table 2, Figure 4), when compared to that of control group.

CD68^+^ macrophages were usually outnumbered by CD163^+^ macrophages as well as CD206^+^ macrophages at the same stage of CML; however, there was no difference between the positive expression of CD163 and CD206 (*P* > 0.01).

### 3.5. The Positive Percentage Ratios of CD163 and CD206 to CD68 Were Upregulated with the Progression of CML

**Figure 5:**
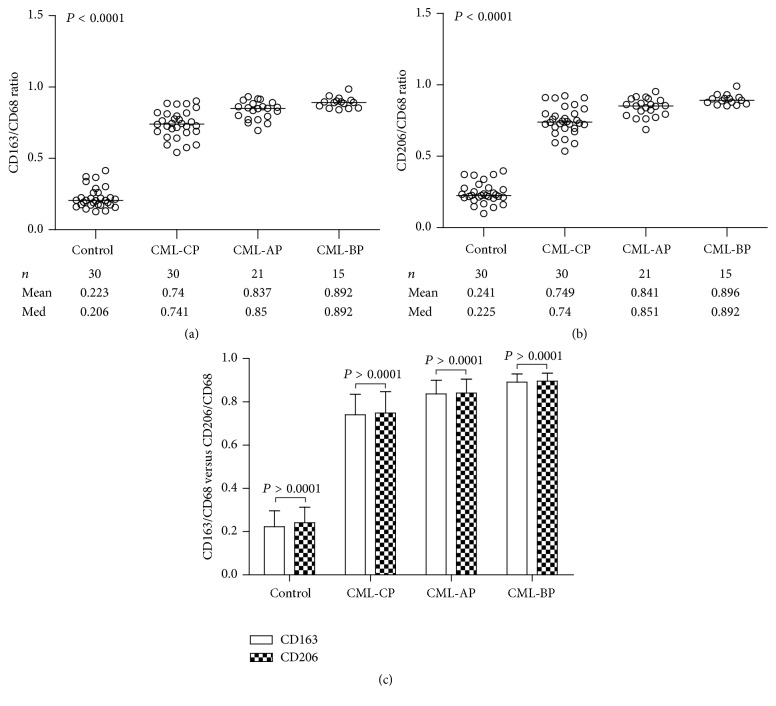
Analysis of infiltrating macrophages in BMB samples of CML patients. The proportions of CD163-positive (a) and CD206-positive (b) cells among CD68-positive cells associated with the different phase of CML. There was no difference between the positive expression ratio of CD163 and CD206 to CD68 (c) at the same phase.

The percentage ratios of CD163+ and CD206*+ macrophages* to CD68*+ macrophages* in CML-CP, CML-AP, and CML-BP were significantly higher than those in control group, respectively (Figures 5(a) and 5(b)). These data imply that activation of macrophages in BMB samples towards the M2-like phenotype associated with the phase's development of CML. However, there was no difference between the proportion of CD163 and CD206-positive cells to CD68-positive cells at the same stage (Figure 5(c)). It was decreased in the released patients with CML-CP (45.77%), CML-AP (66.90%), and CML-BP groups (78.27%) (Table 2). In contrast to the control group, there existed statistically significant (*P* < 0.01).

## 4. Discussion

Macrophages, a remarkably heterogeneous population, played an important role in tumor biology from early carcinogenesis to tumor progression including metastases [9–11]. However, the role of macrophages in CML remains to be elucidated. To examine whether expression of macrophages markers CD68, CD163, and CD206 associated with the CML development, immunohistochemical staining was performed. In this work we provide evidence of its counts and phenotype in BMB samples of CML patients. Further we show that a high frequency of CD68^+^, CD163^+^, and CD206^+^ macrophages associated with the CML progression.

CD68 expressed on all macrophages has been widely used as a pan-macrophage marker; CD163 and CD206 are particular in the M2 phenotype [14, 19]. Our data show that the higher expression of CD68, CD163, and CD206 in BMB samples is a remarkable phenomenon during the transformation of CP to AP and BP in CML patients in contrast to those in control group. Moreover, in this study, the change of the macrophages phenotype was determined in CML development, which indicates that BM microenvironments imbalance, in CML patients, was towards M2-like macrophages. This revealed that leukemia cells' survival and disease progression were associated with high counts of macrophages, particular in M2-like macrophages. Numerical increase in TAMs may be possibly related to enhancing phagocytic activity regarding the degradation of leukemia cells as well as to immune escape [17, 20]. The previous reports have indicated that the CD68^+^ macrophages were increasing in BM of CML [21, 22], but the significance of CD163 and CD206 is expressed on BMB samples and it is associated with phases development of CML patients that has not been explored.

Markers of the M2 phenotype include CD163, CD184, CD204, CD206, CD209, receptors SR-A and M60, and so forth [23, 24]. The proteins of both CD163 and CD206 are distributed in the membranes. However, there are slight differences between CD206-positive and CD163-positive M2 macrophages. M2 macrophages appear to be a heterogeneous population including M2a, M2b, and M2c in response to stimuli provided by local microenvironment. M2a subpopulations express high levels of CD206 upregulated by IL-4 or IL-13. M2b is induced by immune complexes and TLRs or IL-1R. The roles of M2a and M2b are immunoregulatory. M2c is induced by IL-10 and glucocorticoids. M2c has the expression of CD163 and takes part in tissue remodeling [25]. We observed that CD163+ and CD206+ M2-like TAMs increased as the stages progressed in BMB samples of CML patients. But it still not completely understood their functional roles during phenotype changes and how M2-like macrophages cross talk to leukemia cells.

Leukemia cells are largely controlled by specific local microenvironments (i.e., “niches”) and require a suitable microenvironment to maintain their growth [26]. However, the factors and mechanisms providing by bone marrow (BM) microenvironment are not fully understood [27, 28]. BM microenvironment comprised a variety of immune or nonimmune stromal cells like macrophages, adipose cells, mesenchymal stem cells and reticular cells, and so forth. Macrophages are the hot spots of cancer research [7–11]. TAMs are highly plastic dictated by their microenvironment conceptualized into two opposing but complementary activation states: either proinflammatory M1-like TAMs or anti-inflammatory M2-like TAMs [29]. M2-like TAM are alternatively activated and produce anti-inflammatory cytokines like IL-4, IL-10, IL-13, transforming growth factor-b (TGF-b), and various chemokines to turn off damaging immune system activation and get involved in proliferation and survival of leukemia stem cell (LSCs), progression and metastasis, facilitating angiogenesis and lymphangiogenesis, matrix remodeling, and tumor cell-motility [30, 31]. Our results showed that M2-like macrophages were the major components of infiltrating TAMs that orchestrate various aspects of leukemia cells. In the beginning of CML, TAMs were the phenotype of the proinflammatory M1-like TAMs taking part in the antitumor. With the CML development, BM inflammation microenvironment induced the higher numbers of total TAMs, especially in M2-like TAMs. The balance of M1-like TAMs over M2-like TAMs was shifted towards the anti-inflammatory M2-like TAMs. Nonetheless upregulation of CD163 and CD206 is specific to M2-like TAM adaptation towards inflammation shown in BM of CML patients. The numbers of TAM, especially M2 positively phenotype, correlate with the differential stages of disease development of CML patients, which associated to the complex and multidirectional interaction between BM microenvironment and leukemia cells. We speculated that there exists a vicious connection between macrophages and cancer.

M2-like macrophages as their high expression get involved in the suppression of antitumor immune responses [7–11, 18–20]. Some articles described that M2-like macrophages can be converted to M1-like cells and reduced the immunosuppressive effects following IFN-*γ* or other agent's treatment [17, 31–35]. It is suggested that new possible treatment strategies targeting M2-like macrophages in CML are round the corner.

TAM infiltration density in the release group was decreased in BMB samples of CML. In contrast to the control group, there existed statistical significance. It is suggested that the pathogenic factors involved in promoting leukemia cell exaggerated proliferation, differentiation blocked, and apoptosis resistance still existed, so the therapy strategy of CML needed to combine with modification bone marrow (BM) homeostasis [5, 36].

Macrophages are described as part of the mononuclear phagocyte system [37]. The developmental origin of macrophages has bone marrow hematopoiesis derived monocytes, yolk sac macrophages, and fetal monocytes [38–40]. Each tissue throughout the body has its own macrophages including resident macrophages and circulation macrophages [41]. In response to inflammation, infection, and tissue injury, macrophages are recruited into lesions [42, 43]. We observed the expansion of macrophages in BM of CML patients, but whether monocyte recruitment or proliferation of resident or migration of other tissue's registration macrophages needs to be further identified.

## 5. Conclusion

The present study thus highlights the importance of positive expression of CD68, CD163, and CD206 increased in BM localization during chronic to blast phase transition of CML. The positive percentage ratios of CD163 and CD206 to CD68 are roaring upregulation, so targeting M2-like macrophages may help designing therapeutic strategies for CML. However, the reasons that resulted in accelerated macrophage expansion and increased M2-macrophages require in-depth studies.

## Figures and Tables

**Table 1:** Characteristics of the CML patients in this study.

	CML-CP	CML-AP	CML-BP
Number of cases	30	21	15
Gender			
Male	18	13	8
Female	12	8	7
Age, years			
Median (range)	47.60 (18–76)	48.05 (20–79)	47.67 (27–78)
Outcomes			
Released CML-CP	30		
Released CML-AP		15	
Relapsed CML-AP		3	
Released CML-BP			9
Relapsed CML-BP			4

**Table 2:** The positive percentages of the expression among CD68, CD163, and CD206 in BMB samples of CML patients with different phases (percentage, mean ± SD).

Group	Cases	CD68	CD163	CD206	CD163/CD68 (%)	CD206/CD68 (%)
Control	30	12.39 ± 2.17	2.71 ± 0.82	2.94 ± 0.79	21.90	23.73
CML-CP	30	27.03 ± 3.90	19.91 ± 3.33	20.71 ± 3.47	73.68	76.62
CML-AP	21	44.64 ± 4.84	37.26 ± 4.70	38.57 ± 5.12	83.63	86.40
CML-BP	15	66.98 ± 6.28	59.97 ± 6.79	61.51 ± 6.48	89.23	91.83
Released CML-CP	30	19.73 ± 3.43	8.73 ± 2.00	9.03 ± 2.01	44.37	45.77
Released CML-AP	15	25.77 ± 4.83^*∗*^	17.23 ± 3.24	17.24 ± 3.27	66.84	66.90
Relapsed CML-AP	3	48.80 ± 8.15	41.67 ± 7.08	41.67 ± 7.03	85.38	85.39
Released CML-BP	9	43.49 ± 4.80	34.11 ± 4.95	34.04 ± 5.06	78.44	78.27
Relapsed CML-BP	4	78.00 ± 4.49	66.85 ± 5.23	66.85 ± 5.17	85.71	85.71

(1) Data are expressed as percentage of positive staining. (2) Asterisks (*∗*) indicate that the expression levels of CD68 did not exist statistically significant with respect to released patients with CML-AP group and CML-CP (*P* > 0.01). There was statistical significance among the other group and control group (*P* < 0.01).
